# Pharmacological Properties of Four Plant Species of the Genus *Anabasis*, *Amaranthaceae*

**DOI:** 10.3390/molecules28114454

**Published:** 2023-05-31

**Authors:** Zhanybek Shegebayev, Aknur Turgumbayeva, Ubaidilla Datkhayev, Kairat Zhakipbekov, Assem Kalykova, Elmira Kartbayeva, Ahmet Beyatli, Kuanysh Tastambek, Gulmira Altynbayeva, Bassymbek Dilbarkhanov, Aiman Akhelova, Rabiga Anarbayeva, Kulpan Orynbassarova

**Affiliations:** 1 School of Pharmacy, JSC “S.D. Asfendiyarov Kazakh National Medical University”, Almaty 050000, Kazakhstan; zhanybek.shegebayev@gmail.com (Z.S.); u.datxaev@mail.ru (U.D.); zhakipbekov.k@kaznmu.kz (K.Z.); dilbarkhanov.b@kaznmu.kz (B.D.); aiman.akhielova_75@mail.ru (A.A.); 2 Higher School of Medicine, Al-Farabi Kazakh National University, Almaty 050040, Kazakhstan; a.kalykova@gmail.com (A.K.); e.kartbayeva@gmail.com (E.K.); tastambeku@gmail.com (K.T.); 3 Departament of Medicinal and Aromatic Plants, University of Health Sciences, Istanbul 34668, Turkey; ahmet.beyatli@sbu.edu.tr; 4 Ecology Research Institute, Khoja Akhmet Yassawi International Kazakh-Turkish University, Turkistan 161200, Kazakhstan; 5 Department of Biotechnology, M. Auezov South Kazakhstan University, Shymkent 160012, Kazakhstan; 6 Neonatology and Neonatal Surgery Department, JSC “Scientific Center of Pediatrics and Pediatric Surgery”, Almaty 050060, Kazakhstan; altynbayeva.g85@gmail.com; 7 South Kazakhstan Medical Academy, Shymkent 160001, Kazakhstan; rabiga.rm@mail.ru (R.A.); kulpan_ok@mail.ru (K.O.)

**Keywords:** *Anabasis*, Amaranthaceae, traditional use, pharmacological properties, antioxidant

## Abstract

The genus *Anabasis* is a member of the family *Amaranthaceae* (former name: *Chenopodiaceae*) and includes approximately 102 genera and 1400 species. The genus *Anabasis* is one of the most significant families in salt marshes, semi-deserts, and other harsh environments. They are also renowned for their abundance in bioactive compounds, including sesquiterpenes, diterpenes, triterpenes, saponins, phenolic acids, flavonoids, and betalain pigments. Since ancient times, these plants have been used to treat various diseases of the gastrointestinal tract, diabetes, hypertension, and cardiovascular diseases and are used as an antirheumatic and diuretic. At the same time, the genus *Anabasis* is very rich in biologically active secondary metabolites that exhibit great pharmacological properties such as antioxidant, antibacterial, antiangiogenic, antiulcer, hypoglycemic, hepatoprotective, antidiabetic, etc. All of the listed pharmacological activities have been studied in practice by scientists from different countries and are presented in this review article to familiarize the entire scientific community with the results of these studies, as well as to explore the possibilities of using four plant species of the genus *Anabasis* as medicinal raw materials and developing medicines based on them.

## 1. Introduction

The genus *Anabasis* is a member of the family Amaranthaceae (former name: Chenopodiaceae) and includes approximately 102 genera and 1400 species, which are mostly halophytes [1,2]. The genus *Anabasis* is one of the most important families in inhospitable places such as deserts, semi-deserts, and salt marshes [3,4]. *Anabasis* species are often an important source of forage for grazing [5]. Some have been exploited as a commercial supply of lye or potash, while others have served as important fuel sources [6,7]. Since many specimens lack the required characteristics for species identification, it has historically been challenging to identify collections because many members of this family are succulent and late in flowering and fruiting [8,9,10]. The primary quality of these plants is that they are especially resilient to saline environments and environmental stresses [11,12,13]. However, the genus *Anabasis* has attracted the attention of the scientific community due to the characteristics of biologically active secondary metabolites. It is also renowned for being abundant in biologically active compounds, including saponins, sesquiterpenes, diterpenes, and triterpenes, as well as betalain pigments, flavonoids, phenolic acids, glycosides, and glycuronides [14,15,16]. These plants have been used as medicinal herbs [17]. Since ancient times, these plants have been used to treat various diseases. The leaves of the plant are used as an antirheumatic and diuretic [18,19]. The antioxidant activity of plants of this family is also reported [20,21]. Traditionally, they are used for cardiovascular disease, diabetes, and hypertension [22,23,24] but are mainly used for the treatment of the gastrointestinal tract and exhibit antibacterial activity associated with the digestive tract [25,26,27]. It is known that plants and food sources can help fight various types of diseases [28]. Today, up to 80% of the population in developing countries use plants as medicine [29]. Therefore, there is an urgent need to identify and study natural sources with fewer side effects for drug development based on them. Thus, the present study was conducted in order to broadly familiarize the scientific community with the pharmacological activity of various secondary metabolites, identified in the course of studies using various extracts, from representatives of the four most common and well-studied plant species of the genus *Anabasis*, *Anabasis aretioides*, *Anabasis articulata*, *Anabasis aphylla*, and *Anabasis setifera*, as well as *Anabasis salsa,* the effects of which have shown positive medicinal properties.

## 2. Methods

We searched the literature in scientific search engines such as Scopus, Clarivate, MDPI, Wiley Online PubMed, ScienceDirect, and Google Scholar from 1990 to 2022 to study extracts of the genus *Anabasis* as well as its pharmacological properties. *Anabasis articulata, Anabasis aretioides, Anabasis aphylla, Anabasis setifer*, and *Anabasis salsa* were used as keywords, as well as all the names of the pharmacological properties described in this manuscript.

## 3. Traditional Use

*Anabasis aretioides* (Coss & Moq.) is a cylindrical plant widely distributed in the southeastern region of Morocco (Tafilalet) [30]. Since ancient times, this plant has been used to treat various diseases. The leaves of the plant are used as an antirheumatic and diuretic, as well as an antidote for poison [31]. According to studies, A. aretioides has been traditionally used to treat diabetes and hypertension, as well as the associated dyslipidemia [32]. *Anabasis articulata* (Forssk) Moq. is a wild plant that is frequently utilized in Algerian folk medicine to treat dermatitis, kidney infections, fever, headaches, diabetes, and other health issues [33]. It is used alone in decoctions or mixed with other medicinal plants [34]. It is regarded for its larvicidal qualities in addition to its antidiabetic effects [35] and also has hepatoprotective and therapeutic effects against liver damage [36]. *Anabasis aphylla* (haze shrub) has important ecological, economic, and medicinal value [37]. Moreover, *A. aphylla* has a definite distribution in China, Iran [38], Kazakhstan, and Kyrgyzstan [39]. In China, it is mainly distributed in Xinjiang and is mostly used to treat gastrointestinal disorders, diabetes, and rheumatism [40]. *Anabasis setifera* has been used as a medicinal herb in Iran and has been used mainly in the treatment of cardiovascular disease, kidney failure, lung inflammation, and in the prevention of arthritis and atherosclerosis [41]. In scientific works, there are very often references to the widespread use in traditional medicine of *Anabasis salsa*, which grows mainly in the territory of Central Asia, in particular Kazakhstan. *Anabasis salsa* is very popular in folk medicine. An aqueous tincture of the seeds is used to treat paralysis and neuritis, and an alcoholic tincture is used for muscle atrophy and psoriasis. An infusion of the root of *Anabasis salsa* helps well with hypotension and that of dry flower heads with inflammation of the trigeminal nerve. The plant can also be used for other diseases, in particular for sexual weakness, hysteria, physical and mental exhaustion, disorders in the central nervous system, atherosclerosis, mumps, and joint pain. External treatment with decoctions, infusions, and tinctures of *Anabasis salsa* helps with purulent wounds, boils, and eczema. However, unfortunately, at the moment this plant has been little studied from a scientific point of view [42,43].

The chemical composition of the plant product is determined by qualitative chemical analysis using various extraction solvents (Table 1). The choice of method and solvent used for extraction is a particularly important step to obtain the optimal concentration of natural compounds in the extract. It is important to select an efficient extraction method and appropriate work steps to ensure high throughput and improved stability of extractable compounds. To extract phenolic compounds from *Anabasis aretioides*, *Anabasis articulata*, *Anabasis aphylla*, and *Anabasis setifera*, various traditional methods were used, such as maceration with polar and non-polar solvents (such as methanol, ethanol, dichloromethane, ethyl acetate, 10% H_2_O_2,_ oil, and water), Soxhlet extraction, also extracts of butanol and hexane.

**Figure 1:**
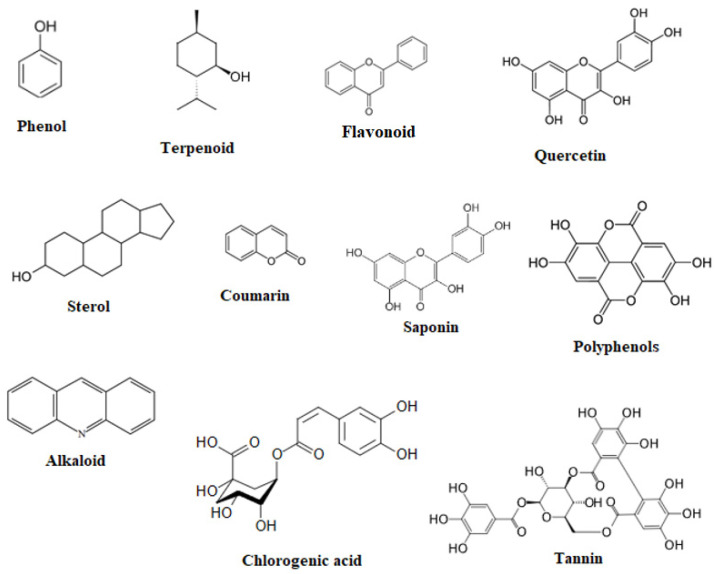
Chemical structure of the main biologically active substances of the genus Anabasis.

Phenolic compounds, saponins, flavonoids, and alkaloids (Figure 1) isolated from *Anabasis aretioides, Anabasis articulata, Anabasis aphylla,* and *Anabasis setifera* were analyzed by HPLC-DAD, UV–Vis detector, *HPLC-UV, GC-MS,* ^1^H NMR, ^13^C NMR, MS, TLC, RPLC-MS/MS, and Folin–Ciocalteu colorimetry. These methods are some of the most common methods of analysis for phenolic compounds (flavonoids, tannins) in plants.

## 4. Pharmacological Activities

### 4.1. Antioxidant Properties

#### 4.1.1. *Anabasis aretioides*

Aqueous and methanolic macerated extracts of *Anabasis aretioides* in a study by Senhaji et al. (2020) [44] showed good antioxidant activity assessed in vitro with five tests: H_2_O_2_ (hydrogen peroxide removal), DPPH (activity purification method), ABTS (trolox equivalent antioxidant capacity assay), FRAP (iron/antioxidant reduction power), and reduction capability assay. The aqueous macerated extract had the highest hydrogen peroxide activity (7.84 ± 0.44%). The macerated methanol extract performed best on the other four antioxidant activity tests, reducing DPPH with an IC_50_ of 52.91 ± 0.24 mcg/mL and having the highest radical scavenging capacity of ABTS•+ (48.99 ± 1.316 µg TE/mg E); it also showed the highest antioxidant activity in the FRAP test (99.73 ± 3.570 µg TE/mg E) and reducing ability (72.176 ± 0.540 µg AAE/mg E) (Table 2).

In another study by Berrani et al. (2019) [45], the antioxidant activity of different parts of *Anabasis aretioides* was studied and compared with the free radical scavenging activity of DPPH gallic acid. Different parts of the plant showed variability in antioxidant activity. The percentages of inhibition were 70.50% for the seeds, 80.79% for the roots, and 78.29% for the aerial parts. As a result, aerial parts and roots have a relatively high antioxidant capacity, and their respective IC_50_ values are: 0.61 ± 0.01 mg/mL and 0.40 ± 0.00 mg/mL compared to seeds with IC_50_ = 1.13 ± 0.01 mg/mL (Table 2). The extracts’ thin liquid chromatography identified several substances that could reduce the DPPH radicals [46]. The results show that the roots have a significant antioxidant effect compared to other parts of the plant with a value of 1.39 ± 0.07 mg/mL, followed by aerial parts with 0.77 ± 0.11 mg/mL. The seed portion showed a low antioxidant effect (0.539±0.07 mg/mL) [47]. The results of ABTS decolorization analysis showed a similar effect, with roots being the most active at 28.27 ± 8.29 mg TR/g [48].

Extracts of *Anabasis aretioides* in studies by Sun and Shahrajabian (2023) [49] showed an antioxidant effect on the DPPH radical, and their activity was compared with various positive controls: the synthetic antioxidant BHA, ascorbic acid, and quercetin. The acetone extract showed the best free radical scavenging activity [50]. It is also reported that methanol and ethyl acetate extracts showed the same activity. Compared to the positive control, all extracts showed lower antioxidant activity (significant differences, *p* < 0.05) [51].

The presence of significant charges of phenolic compounds can be used to explain the antioxidant activity of *Anabasis aretioides* extracts. These substances function as metal chelating potentials, hydrogen donors, reducing agents, and singlet oxygen emitters [52,53].

#### 4.1.2. *Anabasis articulata*

In studies to determine the antioxidant properties of stem and root extracts of *Anabasis articulata*, studies were carried out with various methods, including total antioxidant activity, reducing ability, DPPH radical scavenging activity, and β-carotene bleaching assay. In a study by Atik-Bekkara et al. (2013) [54], the stems show higher levels of phenolic compounds (25.48 mg GAE/g dw) compared to the roots (19.85 mg/g dw) (Table 2). There was no discernible difference between the two portions regarding the plant’s total antioxidant activity. In the tested models, all extracts showed varying degrees of antioxidant qualities. The crude root extract showed the highest activity in reducing ability with an IC_50_ of 0.36 mg/mL, DPPH radical scavenging with an EC_50_ of 0.57 mg/mL, and inhibition of β-carotene oxidation with an EC_50_ of 0.22 mg/mL [55].

Methanol and ethyl acetate extracts of *Anabasis articulata* also show good antioxidant properties. The IC_50_ of methanol extracts (3.200 ± 0.088 mg/mL) has been reported to show good DPPH radical scavenging activity in contrast to the ethanol extract (4.9 mg/mL) [56]. This value remains more or less comparable to that of ascorbic acid (5 mg/mL) and lower than that of quercetin (5.9 mg/mL) [57]. The FRAP iron reduction method showed that the methanol extract has good antioxidant activity (0.100 ± 0.035) and is superior to other extracts. This value is similar to quercetin (0.100 ± 0.104 mg/mL) [58].

Almost all of the extracts from Anabasis articulata have the capacity to inhibit DPPH free radicals, according to a study on the antioxidant activity of raw and purified fractions of the plant. The n-hexane fraction and crude extract, however, demonstrated notable free radical inhibition with IC_50_ values of 45 g/mL and 90 g/mL, respectively [59]. The IC_50_ value of ascorbic acid used as a standard was 32 µg/mL, while the methanol fraction and n-hexane extract showed free radical inhibition with IC_50_ values of 75 µg/mL and 71 µg/mL, respectively (Table 2) [60].

The reduction of Mo(VI) to Mo(V) by antioxidant compounds and the formation of the green phosphate/Mo(V) complex at acidic pH were used to determine the total antioxidant capacity of the alkaloid extracts of Anabasis articulata. It has been discovered that the antioxidant capacities of all alkaloid extracts varied. Tetravalent alkaloids showed the highest total antioxidant capacity (14.74 ± 0.22 mg AAE/g DM), followed by pure basic alkaloids (8.72 ± 0.46 mg AAE/g DM) [54]. The EC_50_ value of each sample was determined to compare the antiradical activity of these extracts. In a proxy test, low EC_50_ values indicate high antioxidant activity. In another study, pure basic alkaloids also showed higher antiradical activity (1.24 ± 0.16 mg/mL), followed by total basic alkaloids (1.38 ± 0.03 mg/mL). The radical scavenging capacity of DPPH was low at 5.35 ± 0.02 mg/mL for the total alkaloid extract, while the tetravalent alkaloid extract had no EC_50_ value under the tested operating conditions. These EC_50_ values were higher than those of ascorbic acid (0.09 ± 0.002 mg/mL) and BHA (0.05 ± 0.003 mg/mL) and were used as positive controls [61]. All samples of alkaloids showed an inhibitory effect on the discoloration of β-carotene at various concentrations by scavenging linoleic acid free radicals. The extracts of total alkaloids and the main alkaloids showed good antioxidant activity with values of 0.35 ± 0.17 and 0.37 ± 0.08 mg/mL, respectively, while the value of the extract of pure basic alkaloids was 0.94 ± 0.03 mg/mL. However, the extract of tetravalent alkaloids showed a high value of 2.31 ± 0.56 mg/mL. These results were lower than for gallic acid (3.22 ± 0.02 mg/mL) but higher than for BHA (0.03 ± 0.005 mg/mL). Compared to other data, total alkaloids reduced the degree of destruction of β-carotene by reacting with the free radical of linoleate at EC_50_ = 1.67 ± 0.22 mg/mL [20].

#### 4.1.3. *Anabasis aphylla*

Studies with two different methods—the DPPH free radical scavenging assay and the β-carotene/linoleic acid assay—have demonstrated the antioxidant capacity of ethyl acetate and methanol extracts of *Anabasis aphylla*. The extracts did not show antioxidant activity in the DPPH method [62]. In the β-carotene/linoleic acid method, the level of antioxidant activity of a substance was determined by measuring the oxidation products of linoleic acid, which simultaneously attack β-carotene, resulting in discoloration of its characteristic yellow color [63,64]. With increasing concentration, a rise in antioxidant activity was seen in ethyl acetate and methanol extracts. The antioxidant activity of ethyl acetate and methanol extracts at high concentrations (100 mg/mL) reached significant levels, 81.8% and 79.3%, respectively [65].

The presence of phenolic hydroxyl or methoxyl groups, flavone hydroxyl groups, keto groups, free carboxyl groups, and other structural characteristics may contribute to antioxidant activity [66].

**Table 2:** Antioxidant activities of plant species of the genus *Anabasis*.

№	Extracts	H_2_O_2_ Scavenging Activity (%)	DPPH IC_50_ (µg/mL)	ABTS (µg TE/mg E)	FRAP (µg TE/mg E)	β-Carotene Test EC_50_ (mg/mL)	RP (µg AAE/mg E)	Reference
*Anabasis aretioides*								
1	Decocted	4.52 ± 0.69	1117.67 ± 0.27	1.45 ± 0.027	2.896 ± 0.209	−	1.727 ± 0.047	[44]
2	Infused	5.96 ± 0.19	3704.33 ± 5.97	0.69 ± 0.093	0.456 ± 0.045	−	0.204 ± 0.031	[44]
3	Macerated	7.84 ± 0.44	2704.33 ± 1.91	1.56 ± 0.006	1.790 ± 0.008	−	0.539 ± 0.081	[44]
4	Macerated methanol	5.32 ± 0.23	52.91 ± 0.24	48.99 ± 1.316	99.736 ± 3.570	−	72.176 ± 0.540	[44]
5	Methanol	3.81 ± 0.26	59.65 ± 1.67	39.10 ± 0.572	79.214 ± 2.031	−	59.954 ± 1.505	[44]
6	Ethyl acetate	3.65 ± 0.80	76.08 ± 1.28	48.06 ± 0.93	83.743 ± 6.346	−	63.480 ± 3.701	[44]
7	Chloroform	2.81 ± 0.43	863.60 ± 10.49	31.89 ± 1.17	50.199 ± 1.341	−	23.376 ± 1.601	[44]
8	Petroleum ether	4.91 ± 0.38	515.53 ± 1.39	10.61 ± 1.528	24.601 ± 1.466	−	4.640 ± 0.099	[44]
9	Ascorbic acid	14.35 ± 0.002	0.17 ± 0.02	−	−	−	−	[44]
10	BHT	−	1.59 ± 0.13	−	−	−	−	[44]
11	Trolox	−	1.75 ± 0.09	−	−		−	[44]
12	Acetone extract	23.81 ± 2.13^a^	47.71 ± 1.13	−	−	−	−	[45]
13	Methanol extract	26.98 ± 2.99 ^a^	79.15 ± 4.23	−	−	−	−	[45]
14	Chloroform extract	29.28 ± 5.04 ^a^	86.73 ± 10.68	−	−	−	−	[45]
15	Ethanol extract	28.72 ± 3.03 ^a^	65.08 ± 1.98	−	−	−	−	[45]
16	Ethyl acetate	45.49 ± 3.84 ^b^	72.15 ± 1.04	−	−	−	−	[45]
17	BHA extract	24.13 ± 7.32 ^a^	2.61 ± 0.13	−	−	−	−	[45]
18	a-Tocopherol	32.44 ± 5.87 ^a^	−	−	−	−	−	[45]
*Anabasis articulata*								
19	Ethyl acetate and *n*-hexane (5:95)	−	71.31 ± 0.45	72.45 ± 0.79	−	−	−	[54]
20	Ethyl acetate and *n*-hexane (10:90)	−	71.34 ± 0.65	59.48 ± 0.27	−	−	−	[54]
21	Ethyl acetate and *n*-hexane (10:90)	−	34.11 ± 0.87	64.78 ± 0.69	−	−	−	[54]
22	Ethyl acetate and *n*-hexane (30:70)	−	50.36 ± 0.88	67.86 ± 0.95	−	−	−	[54]
23	Oil fraction	−	78.24 ± 0.32	64.52 ± 0.39	−	−	−	[54]
24	Ascorbic acid	−	78.64 ± 0.63	78.35 ± 0.73	−	−	−	[54]
25	Total alkaloids	−	5.350 ± 0.022	−	−	0.353 ± 0.175	−	[54]
26	Basic alkaloids	−	1.380 ± 0.037	−	−	0.372 ± 0.086	−	[60]
27	Tetravalent alkaloids	−	−	−	−	2.313 ± 0.557	−	[60]
28	Pure basic alkaloids	−	1.242 ± 0.168	−	−	2.313 ± 0.557	−	[60]
29	Ascorbic acid	−	0.090 ± 0.002	−	−	−	−	[60]
30	BHA	−	0.054 ± 0.003	−	−	0.028 ± 0.005	−	[60]
*Anabasis aphylla*								
31	Ethyl acetate extracts	−	−	−	−	79.3 ± 0.083	−	[62]
32	Methanolic extract	−	−	−	−	81.8 ± 0.005	−	[62]
*Anabasis setifera*								
33	Acetone extract		28.62 ± 0.02			21.08 ± 0.02		[41]
34	Methanolic extract		24.87 ± 0.06			24.31 ± 02		[41]

### 4.2. Antimicrobial Properties

#### 4.2.1. *Anabasis aretioides*

Various extracts from the aerial part of *Anabasis aretioides* (ethyl acetate, methanol, and macerated methanol) have antibacterial activity against six different strains of bacteria. The extracts showed good efficacy against all strains, including both Gram-positive (*Staphylococcus aureus* CECT976, *Bacillus subtilis* DSM6633, *Listeria innocua* CECT 4030) and Gram-negative bacteria (*Escherichia coli* K12, *Proteus mirabilis*, *Pseudomonas aeruginosa* CECT118). In particular, the ethyl acetate extract showed the highest inhibitory effect against Staphylococcus aureus (13.5 mm), while the methanol and macerated methanol extracts showed the lowest minimum inhibitory concentration for *Proteus mirabilis* (3.125 mg/mL) (Table 3). The studies were carried out using various methods, including the disk diffusion method in an agar medium and the determination of minimal inhibitory concentrations and bactericidal concentrations [19,44].

Previous studies on *Anabasis aretioides* reported that serial dilution testing in 96-well microplates yielded minimum inhibitory concentration (MIC) values ranging from 7.81 to 31.25 mg/mL. The extract from the seed and aerial parts had a similar MIC value of 7.81 mg/mL and demonstrated an antibacterial activity. Plant seeds were more effective against *S. enterica* and *P. aeruginosa* and aerial parts against two strains, *S. enterica* and *S. aureus*. The MBC values of the MeOH extracts from the various parts were equal to or close to the MIC, indicating strong bactericidal activity. No differences were observed between these strains in MIC and MBC values for all parts of *Anabasis aretioides* [45,67].

The antibacterial activity of *Anabasis aretioides* extracts may be related to phenolic compounds present in plant extracts [68,69]. Gram+ bacteria have been observed to be more sensitive to A. aretioides extracts than Gram-. Gram-negative bacteria have a complicated cell membrane that limits the entry of antibiotic substances, obstructing access to the cell membrane. Lipopolysaccharides’ strong hydrophilicity prevents larger hydrophilic solutes from passing through the porins, preventing this from happening [70,71].

#### 4.2.2. *Anabasis articulata*

The good antimicrobial potential of *Anabasis articulata* has been observed using the disk diffusion method for an extract of pure basic alkaloids. Extracts of total alkaloids, pure basic alkaloids, and tetravalent alkaloids showed no antibacterial activity, with the exception of against *M. luteus* and *Pseudomonas aeruginosa*. This effect was very marked against Gram-positive pathogens, especially M. luteus and L. monocytogenes, which showed the largest IZD at 400 mg/mL of the pure basic alkaloid extract (16 and 20 mm, respectively), followed by *B. subtilis* with an IZD of 14. 25 mm. For *C. albicans*, the extract of tetravalent alkaloids at a concentration of 400 mg/mL showed the smallest zone of inhibition (8 mm) (Table 3) [20,72].

In other studies, a methanolic extract of *Anabasis articulata* at 25 mg/mL was found to exhibit a higher degree of antimicrobial activity against *Pseudomonas aeruginosa* than at 50 mg/mL, probably due to a synergistic effect. At the same time, no effect was found for Bacillus subtilis, in contrast to the methanol control group, where the colonies spread normally.

The results of the study by Al-Joufi et al. (2022) [60] showed that the n-hexane (oily) fraction had the largest ZI against all tested strains: *S. dysentery*, *E. coli*, and *S. typhi*, of 20, 24, and 16 mm, respectively. The broad-spectrum antibiotic ampicillin was used as a positive control.

#### 4.2.3. *Anabasis aphylla*

Extracts of four different concentrations of *Anabasis aphylla* tested for antimicrobial activity using disk diffusion assay against microorganisms such as *Staphylococcus aureus* (PTCC 1764), *Enterococcus faecalis* (PTCC 1394), *Bacillus polymyxa* (ATCC 10401), *Pseudomonas aeruginosa* (CIP A22), *Salmonella typhi* (PTCC 1609), and *Proteus mirabilis* (OXK PTCC 1076) and fungi such as *Aspergillus niger* (PTCC 5223) and *Candida albicans* (PTCC 5027) showed that the n-butanol fraction at a concentration of 100 mg/mL has the maximum antibacterial activity against Enterococcus faecalis (13 mm) and Proteus mirabilis (18 mm) and antifungal activity against *Aspergillus niger* (20 mm), while the 100 mg/mL ethyl acetate fraction showed maximum antibacterial activity against *Proteus mirabilis* (18 mm) and antifungal activity against *Candida albicans* (19 mm). All of these fractions exhibited no antibacterial activity at a 1 mg/mL concentration. Aqueous extracts had antibacterial activity at 25 and 100 mg/mL. Strong antifungal activity was seen against *Aspergillus niger* (14 mm) and *Candida albicans* (12 mm) in aqueous extracts at a dosage of 0.1 mg/mL, as well as antibacterial activity against *Salmonella typhi* (10 mm) and *Proteus mirabilis* (10 mm) (Table 3) [65,73].

Additionally, a study of the antimicrobial activity of six phenolic compounds (1-(2-hydroxy-4,6-dimethoxyphenyl)ethanone (**1**), 3,4-dihydroxycinnamic acid (**2**), 4-hydroxy-3-methoxybenzoic acid (**3**), 2-hydroxybenzoic acid (**4**), 3,4-dihydroxycinnamic acid methyl ester (**5**), and 4-hydroxybenzoic acid pentadecane ester (**6**)) of *Anabasis aphylla* obtained by fractionation of an ethyl acetate extract from aerial parts, tested for their minimum inhibitory effect (MIC) and median inhibitory concentration (IC_50_) using microdilution-MTT assay for antimicrobial activities against one Gram-positive bacterium, *Bacillus subtilis*, three Gram-negative bacteria, *Agrobacterium tumefaciens*, *Pseudomonas lachrymans*, and *Xanthomonas vesicatoria*, and one species of yeast, *Candida albicans*, found that apart from the last compound, which did not show activity against any of the tested microorganisms, the remaining compounds exhibit selective inhibitory activity. It should be noted that this is the first report on the antimicrobial activity of phenolic compounds isolated from *Anabasis aphylla* [74,75].

The studies of Du et al. (2009) [74] showed that crude ethanol extracts and their various polar fractions from the aerial parts of *Anabasis aphylla* exhibited antibacterial activity against six bacterial strains, *Agrobacterium tumefaciens*, *Bacillus subtilis, Escherichia coli, Pseudomonas lachrymans, Staphylococcus haemolyticus*, and *Xanthomonas vesicatoria*, as well as antifungal activity against eleven species of fungi, *Alternaria solani, Bipolaris maydis, Rhizoctonia ceramicis, R. solani, Fusarium graminearum, F. oxysporum f* sp. *cucumerinum, F. oxysporum f* sp. *niverum, F. vasinfectum, Venturia pirina, Leptosphaeria biglobosa*, and Magnaporthe grisea, in vitro. Both the ethyl acetate fraction and the n-butanol fraction of this plant species had stronger antimicrobial activity than the petroleum ether fraction and the aqueous fraction. The results showed that active antimicrobial compounds can be associated with alkaloids of moderate polarity, are slightly alkaline, and easily form salts with acid in *Anabasis aphylla*.

**Table 3:** Antibacterial activity of plant species of the genus *Anabasis*.

№	Extracts	Concentration (mg/mL)	*E. coli*	S.a	P.a	C.a	Ref.
*Anabasis articulata*							
1	*Saponin* *alkaloids*	5	17.8 16.3	10.1 12.4	21.1 13.3	13 -	[72]
2	*Saponin* *alkaloids*	2.5	15.2 13.2	8.5 12	16.1 10.8	10.5 -	[72]
3	*Saponin* *alkaloids*	1	10.3 13.3	8.1 7.6	12.5 8.5	9.3 -	[72]
4	*Saponin* *alkaloids*	0.5	7 -	10.9 -	10.2 7.4	8.8 -	[72]
5	Total alkaloids	80	10	10	5	10	[72]
6	Tetravalent alkaloids	400	20	>20	>20	20	[72]
7	Pure basic alkaloids	400	0.781	3.125	25	>100	[72]
*Anabasis aphylla*							
8	Methanol	100	-	8	11	17	[65]
9	Ethyl acetate	100	-	10	14	19	[65]
10	n-Butanol	100	-	10	10	18	[65]
11	Water	100	-	0	7	12	[65]
12	1-(2-hydroxy-4,6-dimethoxyphenyl)-ethanone	-	-	-	-	100.0	[65]
13	3,4-dihydroxy cinnamic acid tetracosyl ester	-	-	-	-	200.0	[65]
14	4-hydroxy 3-methoxy benzoic acid	-	-	-	-	200.0	[65]
15	2-hydroxy benzoic acid	-	-	-	-	100.0	[65]
16	3,4-dihydroxy cinnamic acid methyl ester	-	-	-	-	200.0	[65]
*Anabasis aretioides*							
17	Macerated methanol	-	-	-	-	-	[44]
18	Methanol	-	-	-	-	-	[44]
19	Ethyl acetate	100	10.5	13.5	8	-	[44]
20	Chloroform	100	11.5	11.5	8	-	[44]

### 4.3. Antiangiogenesis Effect

Abdulsahib et al. (2016) [76] discovered the potential antiangiogenic antioxidant effect of *Anabasis articulata* stem extracts. From the articulated stems of *Anabasis articulata*, a powder was obtained, which was successively treated with petroleum ether, chloroform, methanol, and water using the cold maceration method. 1,1-diphenyl-2-picrylhydrazyl free radical scavenging assay was used to analyze the antioxidant properties. All four extracts (ethanol, butanol, ethyl acetate, and methanol) showed strong inhibition of microvessel growth in the rat aorta assay compared to the negative control (*p* < 0.001), but the methanol extract showed the highest percentage of antiangiogenic activity. Methanolic extracts of *Anabasis articulata* stems showed a significant dose-dependent antiangiogenic effect with an IC_50_ of 18.27 µg/mL. In addition, the methanolic extracts show significant free radical scavenging activity (*p* < 0.05) with an IC_50_ of 94.7 µg/mL. Methanolic extracts of *Anabasis articulata* stems showed the best and most significant antiangiogenic activity, as well as significant free radical scavenging activity.

An in vivo study of the stems of *Anabasis articulata* also showed a good antiangiogenic effect. The stems of *Anabasis articulata* were sequentially extracted with four types of solvents of different polarity. As a result, it was found that the zones of inhibition ranged from 7 to 10 mm. FT-IR has shown that some of the recognized functional groups may relate to flavones, coumarins, alkaline compounds, saponins, and tannins. Scopoletin, 2-methoxy-4-vinylphenol, glycine, and 1,2-dimethyl-piperidine were found in the methanol extract. These are substances that have the ability to block the VEGF receptor, thereby inhibiting angiogenesis [76].

### 4.4. Gastroprotective Effect

The new triterpenoid 3β,20α-dihydroxy-30-norolean-12-en-23,28-dioic acid, isolated from the aerial parts of *Anabasis articulata* (Forssk) Moq, together with 3β-hydroxy-23-aldehyde-lup-20-en-28-oic acid, possesses antiulcer activity. The methylene chloride fraction has strong cytotoxic activity against tumor cell lines HepG-2 (6.9 µg/mL) and HCT-116 (5.5 µg/mL) compared to 5-fluorouracil (7.9 µg/mL). Treatment of rats with indomethacin-induced ulcers with an ethyl acetate fraction (400 mg/kg, po/day) resulted in a decrease in the ulcerative index (0.18) and a strong inhibition of the percentage of ulcers (84.86%) compared with ranitidine. The compound showed a high rate of docking against the H+/K+-AT phase of the stomach [77].

### 4.5. Anti-Inflammatory Activity

Bioguided fractionation of *Anabasis setifera* Moq. demonstrates anti-inflammatory activity against the carrageenan model of paw edema in rats. Based on the percentage inhibition of edema after a 3 h injection of carrageenan, the n-butanol fraction showed promising activity due to a significant (*p* < 0.05) reduction in paw volume of 85.6% compared to control using indomethacin as a reference standard. In addition, the n-butanol fraction significantly (*p* < 0.05) reduced the levels of PGE2 and TNF-α in rat paw edema exudates [34].

### 4.6. Hypoglycemic and Antihyperglycemic Effects

Experiments performed on mice without diabetes and mice with hyperglycemia (mice treated with glucose and alloxan) confirmed the antidiabetic potential of *Anabasis articulata* and found that oral administration of an aqueous leaf extract at a dose of 400 mg/kg reduced glycemia by 29.89% after 6 h (*p* < 0.05), which corresponds to the greatest decrease in blood glucose levels in normoglycemic mice. This dose also reduced blood glucose concentrations in diabetic mice, showing an antihyperglycemic effect of *Anabasis articulata* leaves. Phytochemical screening showed that the aqueous extract contained alkaloids (1.25%) and saponins (1.30%). The results demonstrate that saponin (5 mg/kg) is the active fraction as it restores normal blood glucose levels after 21 days of treatment. The alkaloid fraction did not significantly reduce blood glucose levels. According to the authors, the antihyperglycemic activity of *Anabasis articulata*, due to the release of insulin by the pancreas, has a direct insulinotropic effect, and this may also be due to the insulin-like effect of the active principle (saponin) [34].

The studies carried out by Amin et al. (2022) reported the inhibitory potential of *Anabasis aretioides* methanolic extracts on key enzymes associated with hyperglycemia. In vitro assays showed a moderate inhibitory effect compared to the positive control. The results obtained show that the aerial parts show the most important inhibitory activity with 90.25% and IC_50_ = 2940.59 ± 110.32 μg/g. ml for α-glucosidase, while the roots showed 86.98% inhibition and IC_50_ = 2440.20 ± 84.90 μg/mL for α-amylase. The percentage inhibition of these enzymes increases with increasing concentration [78].

### 4.7. Antidiabetic Effects

The study of the effect of saponin fractions of the alcoholic extract of the aerial parts of the medicinal plant *Anabasis articulata* in comparison with the currently available antidiabetic drug gliclazide (Diamicron) against diabetic complications caused by tissue damage in rats showed an antidiabetic and therapeutic role. Oral administration of the plant modulated the diabetic increase in blood glucose and cortisol levels, suggesting an antihyperglycemic effect of this medicinal plant. It effectively increased the concentration of the hormone insulin and alpha-fetoprotein in the blood. Tumor necrosis factor α (TNF-α) in the blood also significantly decreased. It also effectively reduced blood fructosamine levels to normal levels due to diabetic hemoglobin (Hb) depletion and albumin levels. In addition, the consumption of the plant effectively modulated oxidative damage to liver tissue [79]. According to the researchers, the antidiabetic activity may be related to the components of saponin, and its antihyperglycemic activity is achieved through the release of insulin from the pancreas, that is, it has a direct insulinotropic effect, or it may be due to the insulin-like effect of the active substance (saponin) present in extract [22,80].

A study of the effect of an aqueous extract of *Anabasis aretioides* on blood glucose levels and lipid metabolism in rats with normal diabetes and diabetes induced by streptozotocin was performed using the method inducing the effect of an aqueous extract of the aerial part of *Anabasis aretioides* (A.P.A.E) (5 mg/kg) on blood glucose levels in normal rats and rats suffering from streptozotocin-induced diabetes (n = 6), with an examination of histopathological changes in the liver and pancreas in both normal rats and rats with streptozotocin (STZ)-induced diabetes, in consequence of which it was found that both single and oral administrations (A.P.A.E) (5 mg/kg) caused a significant decrease in blood glucose levels in STZ rats (*p* < 0.0001) [81].

### 4.8. Glaucoma

The methanolic extract of *Anabasis articulata* (AA) showed an antioxidant effect on intraocular pressure in glaucoma rat models. A daily dose of AA extract (50 mg/kg/day) for 6 days significantly reduced intraocular pressure (*p* ˂ 0.05) from (34.23 ± 0.58) to (32.83 ± 1.38) mm Hg. compared with the control group, which experienced an increase in intraocular pressure by being administered a suspension of betamethasone. In another group of rats treated with a dose of 100 mg/kg/day, intraocular pressure significantly decreased from (35.5 ± 1.37) to (31.35 ± 0.40) mm Hg. (*p* ˂ 0.05) after 1 week of treatment. In the group of rats receiving a dose of 150 mg/kg/day, a significant (*p* ˂ 0.001) decrease in IOP from (35.66 ± 0.39) to (31.88 ± 0.74) mm Hg. was shown, starting on the 6th day and continuing until the end of the experiment, reaching (24.53 ± 0.53) mm Hg. (*p* ˂ 0.001) [82].

### 4.9. Antiarthritic Activity

A study to detect the antiarthritic effects of *Anabasis articulata* (AA) in a rat model of arthritis showed good results. Complete Freund’s adjuvant (CFA) was used intradermally (ID) to induce arthritis. AA administration increased body weight (BW), but decreased arthritis index (AI), histopathological parameters, and expression of vascular endothelial growth factor in synovial cells. Compared with the induced group, daily administration of AA significantly reduced the arthritis score in both the treatment (*p* = 0.0034) and prevention (*p* = 0.0023) groups. Compared to the induced group, daily administration of AA in the prophylactic group significantly increased (*p* = 0.0042) BW. In addition, the weight of rats in the AA group had a more significant (*p* = 0.0013) increase [83].

### 4.10. Hepatoprotective Effects

An ethanolic extract of the aerial parts of *Anabasis articulata* showed protective and therapeutic effects on dimethylnitrosamine-induced liver fibrosis similar to the standard drug silymarin. Administration of an ethanol extract of *Anabasis articulata* (100 mg/kg daily for 4 weeks) markedly prevented DMN-induced weight loss in the body and liver. The extract significantly inhibited the increase in hepatic hydroxyproline, NO, and MDA (*p* < 0.05), as well as serum fructosamine and TGF-β1 (*p* < 0.05) induced by DMN, while restoring IL-10 to normal levels in the protective and therapeutic groups. In addition, *Anabasis articulata* prevented depletion of CAT, GR, and GSH levels (*p* ≤ 0.05). At the same time, oral administration of A. articulata extract and silymarin in both protective and therapeutic groups reduced the increase in the activity of liver function enzymes, alanine, aspartate aminotransferases, and gamma-glutamyltransferases, in addition to alkaline phosphatase, and caused a significant increase in serum albumin concentration compared to the DMN group. These data were in close agreement with those obtained for the preparation silymarin [84]. Histopathological studies confirmed the biochemical findings and showed a significant improvement in liver structure [34].

Phenolic compounds contain one (phenolic acids) or several (polyphenols) aromatic rings with hydroxyl groups attached to them. Their antioxidant abilities are related to these hydroxyl groups and phenolic rings. Despite their antioxidant activity, they have many other beneficial properties for human health. However, before the health benefits of these compounds can be attributed, important issues to consider are the absorption, distribution, and metabolism of each phenolic compound in the body. Phenolic compounds are known to exhibit various biological activities such as antimicrobial, antioxidant, and anti-inflammatory properties. Phenolic compounds are mainly classified according to their chemical structures into phenolic acids, flavonoids, tannins, phenolic lignans, and phenolic stilbenes [85,86]

Phenolic compounds have shown antioxidant properties, with their potential being directly related to the type of solvent used in extraction, as well as plant origin, growing conditions, harvest time, and storage conditions [87]. The study of the antioxidant potential of phenolic extracts obtained from plant species *Anabasis aretioides, Anabasis articulata, Anabasis aphylla,* and *Anabasis setifera* is one of the hot topics in the scientific community.

Alkaloids are nitrogen (in the form of a primary, a secondary, or a tertiary amine) and organic molecules, and secondary metabolites of plants, usually containing nitrogen in a ring; about 20% of plant species contain alkaloids [88]. *Anabasis aphylla* methanol extract was produced and, from this extract, an alkaloid with antibacterial and antioxidant effect was isolated. An alkaloid with antibacterial and antioxidant effects was found in the methanol extract of *Anabasis aphylla*.

## 5. Conclusions

Four plant species of the genus Anabasis have a wide and growing range of medicinal potential and have had the majority of their potential actions demonstrated either in vitro or in vivo. Their wide range of therapeutic benefits have also been highlighted by a few clinical investigations. To define and validate the ethnopharmacological profile of these four plant species in the genus Anabasis, more of these types of studies are necessary. Therefore, more research must be carried out using clinical disease models in order to assess and demonstrate the herbs’ effectiveness in treating various ailments. Even if the active compounds are derived from natural sources, using them at high doses to achieve therapeutic benefits may have certain severe adverse effects. In order to perform molecular modification of the active compounds in order to adopt an appropriate therapeutic regimen that can be produced on a commercial scale, future research should be focused on the identification of active constituents in these four plant species of the genus Anabasis, their large-scale synthesis, evaluation of chemical properties, therapeutic evaluation, and toxicity. Another option is to employ these four species of the genus Anabasis as an adjuvant for chemotherapy medications. This would allow for lower dosages of the synthetic drugs and ultimately less severe side effects. The underlying mechanisms of action of these acclaimed medicinal plants should also be revealed through some explanatory mechanistic investigations, which will validate the traditional knowledge related to these cherished medicinal herbs. Strengthening of the extraction techniques already used, medication standardization procedures, and upcoming clinical studies on the health promoting properties of these herbs would all contribute to increasing their practical utility.

## Figures and Tables

**Table 1:** Extracted biologically active compounds from *Anabasis articulata*, *Anabasis aretioides*, *Anabasis aphylla*, *Anabasis setifer*.

Types of Plant	Extracts	Quality of Control	Bioactive Compounds	Pharmacology Activity	Country
*Anabasis* *aretioides*	Maceration methanol	Surface characterization by SEM-EDS	Polyphenol compounds, tannins, coumarins, reducing compounds		Morocco
	Ethanolic, ethyl acetate extracts	Folin–Ciocalteu colorimetric method, 700 and 750 nm spectrophotometer	Phenolic, flavonoid, and α-tocopherol	Antioxidant	Algeria
	Methanolic extract	HPLC-DAD-QTOF-MS	Chlorogenic acid, phenolic compound, hesperidin, quercitrin	Antioxidant and antipathogenic	Morocco
	Aqueous extract		Polyphenols, tannins, saponins, mucilage, sterol, sesquiterpenes, terpenoids, carbohydrates, and glycosides	Antidiabetic	Morocco
	Aqueous extract	HPLC–DAD–ESI–MS	Polyphenol compounds	Antidiabetic	Morocco
	Soxhlet extract, aqueous, and organic extract	Folin–Ciocalteu reagent, 765 nm spectrophotometer	Polyphenols, tannins	Antibacterial and antioxidant	Morocco
*Anabasis articulata*	Methanolic extract and fractions dichloromethane, methanol, and ethyl acetate	HPLC, UV–Vis detector	Phenolic compounds, flavonoids	Antibacterial and antioxidant	Algeria
	Organic extractions	GC/MS	Alkaloids	Antibacterial and antioxidant	Algeria
	Ethanolic extract fractions, water, and partitioned with ethyl acetate	HPTLC, ^1^H NMR and ^13^C NMR, MS, TLC	Saponin	Streptozotocin-induced diabetes Antidiabetic	Egypt
	Aqueous extract		Saponin, alkaloids	Antihyperglycemic, antidiabetic	Algeria
	Methanolic extract	HPLC-UV, GC-MS	Phenolic, flavonoids	Antibacterial, antioxidant, and antidiabetic	Saudi Arabia and Pakistan
	Maceration methanol extract	FT-IR, GC-MS	Scopoletin, glycine and 2-methoxy 4-vinylphenol with minor one of 1, 2-dimethyl piperidine	Antiangiogenic activity	Iraq
	Methanolic extract, fractions	TLC, HPLC, UV–visible spectrophotometry	Phenolic acids	Antioxidative	Algeria
	Butanolic extract		Saponins	Antihyperglycemic	Algeria
*Anabasis aphylla*	Methanolic extract		Alkaloids, saponins, flavonoids, terpenoids, steroids, and sterols	Antimicrobial and antioxidant activities	Iran
	10% H_2_O_2_ extract	HPLC, RPLC-MS/MS, SDS-PAGE electrophoresis	Amino acids		China
	Ethanolic extract	Folin–Ciocalteu reagent	Phenolic compounds	Antimicrobial	Iran
	Ethanolic extract	^1^H NMR and ^13^C	p-Acetyl-phenol 1-O-beta-D-xylopyranosyl-(1-->2)-beta-D-glucopyranoside, together with five known compounds: piceine, isorhamnetin, quercetin, rutin, and isorhamnetin-3-rutinoside		China
	Ethanol (95%) extract and fractions, hot water, and extracted with ethyl acetate	^1^H NMR and ^13^C, TLC	Phenolic compounds	Antimicrobial	China
*Anabasis setifera*	*n*-Butanol fraction	^1^H NMR and ^13^C	α-Amyrin 3-O-glucopyranoside, patuletin 7-O-glucopyranoside, myricitrin, and a new oleanane triterpene saponin derivative, sophradiol 3-O-α-L-1C4-rhamnopyranosyl-(1′′′→4′′)-O-β-D-4C1-galactopyranosyl (1′′→6′)-O-β-D-4C1-glucopyranoside	Anti-inflammatory	China
	Methanol extraction		Phenols, tannins, flavonoids, and saponins	Antioxidant	Iran
	Oil-in-water extraction	^1^H NMR and ^13^C	Saponin		South Africa and Iran
	Ethanolic 70% extract		Lipoidal matter, carbohydrates, proteins, and phenolic compounds	Anti-inflammatory, antidiabetic, antispasmodic, and antimicrobial activities	Egypt

## Data Availability

Not applicable.
